# Letter to the Editor

**Published:** 1986-12

**Authors:** Huw B. Griffith

**Affiliations:** *Senior Neurosurgeon* Frenchay Hospital Bristol


### Letter to the Editor

Sir,
In reply to Mr D. J. Bracey's letter published in your August number. We were of course aware that an English Imtran system had been used (with slow scan) between the Isles of Scilly and Truro. However the IMTRAN system did not derive from this but was developed via their research laboratories at Martlesham. Mr Bracey is to be commended on his fore-sight in making use of the then existing technology in carrying out an important clinical task.

Huw B. Griffith FRCP, FRCS
*Senior Neurosurgeon*
Frenchay Hospital
Bristol

